# Toll-like receptor polymorphisms compromise the inflammatory response against bacterial antigen translocation in cirrhosis

**DOI:** 10.1038/srep46425

**Published:** 2017-04-18

**Authors:** Paula Piñero, Oriol Juanola, Esther Caparrós, Pedro Zapater, Paula Giménez, José M. González-Navajas, José Such, Rubén Francés

**Affiliations:** 1 Instituto de Investigación Sanitaria y Biomédica de Alicante (ISABIAL-Fundación FISABIO), Alicante, Spain; 2 Dpto. Medicina Clínica, Universidad Miguel Hernández, San Juan, Spain; 3 Servicio de Farmacología Clínica, Hospital General Universitario de Alicante, Alicante, Spain; 4 CIBERehd, Instituto de Salud Carlos III, Madrid, Spain; 5 Digestive Disease Institute, Cleveland Clinic Abu Dhabi, UAE

## Abstract

Bacterial translocation is associated with clinically relevant complications in cirrhosis. We evaluated the effect of toll-like receptor polymorphisms in the soluble response against these episodes. Consecutive patients with cirrhosis and ascitic fluid were distributed by TLR2 rs4696480, TLR4 rs4986790, and TLR9 rs187084 single-nucleotide polymorphisms. Lipoteichoic acid, lipopolyssaccharide, bacterial-DNA, pro-inflammatory cytokines and nitric oxide levels were quantified in serum samples. *In vitro* response against specific ligands in variant TLR genotypes was evaluated. One hundred and fourteen patients were included. Variant TLR-2, TLR-4 and TLR-9 SNP genotypes were associated with significantly increased serum levels of LTA, LPS and bacterial-DNA. TNF-α, IL-6 and nitric oxide serum levels were significantly decreased in all variant TLR genotyped patients. Cytokine levels were significantly less upregulated in response to specific TLR-ligands in patients with all variant *vs* wildtype TLR genotypes. Although *in vitro* gene expression levels of all wildtype and variant TLRs were similar, MyD88 and NFkB were significantly downregulated in cells from TLR-variant genotyped patients in response to their ligands. Variant TLR genotypes are associated with an increased circulating antigen burden and a decreased proinflammatory response in cirrhosis. This immunodeficiency may facilitate bacteria-related complications in cirrhosis and enhance TLR targeting for its management.

The translocation of commensal bacterial antigens into blood of patients with decompensated cirrhosis is a frequent event that triggers relevant clinical complications. Several studies have related the transient passage of bacterial products into the systemic circulation of these patients with the exacerbation of the inflammatory response^1^,^2^,^3^,^4^, the prediction of infections^5^, an increased risk of hemodynamic disturbances^4^,^6^ or even death^7^.

Bacterial antigenic products are specifically recognized by a host’s family of receptors, both at the membrane and the intracellular levels. The Toll Like Receptor (TLR) family comprises different receptors that specifically bind unique bacterial products, launching an inflammatory signalling cascade and the mechanisms for these bacterial products clearance^8^,^9^. TLR-2 is the specific receptor for lipoteichoic acid (LTA), a product derived from gram-positive bacteria, which are also able to translocate and differentiate a specific immune response^10^. TLR-4 and TLR-9 are receptors for lipopolysaccharide (LPS)^11^ and bacterial DNA^12^ respectively, two of the most immunogenic bacterial products. Several studies have described the deep effects of endotoxemia or the presence of bacterial DNA on the clinical outcome of decompensated cirrhosis.

Several polymorphisms in these TLR genes have been described and associated with immune alterations or clinical complications in cirrhosis and other inflammatory-related disorders. A polymorphism in the TLR-2 promoter has been linked to an increased risk for spontaneous bacterial peritonitis in cirrhosis^13^. The TLR-4 rs4986790 polymorphism has been found to predispose to infections in cirrhotic patients^14^ and to an LPS hyporresponsiveness^15^. These results have brought the attention into TLRs as potential targets in chronic liver disease^16^. On the other hand, the TLR-9 rs187084 polymorphism has been described to be functionally relevant by downregulating TLR-9 expression^17^.

Therefore, the genetic background may be a relevant potential modulator of the immune response in bacterial-derived complications of cirrhosis. The aim of the present study was to evaluate the incidence of relevant polymorphisms in TLR-2, TLR-4 and TLR-9, specific receptors of bacterial products which are frequently translocated from the gut in patients with cirrhosis.

## Results

### Characteristics of patients

One hundred and fourteen patients with cirrhosis and non-infected AF were included in the study. All clinical and analytical characteristics of patients are detailed in Table 1. Mean age was 60 ± 10 and 68.4% were male. Main etiology of cirrhosis was alcohol, and mean Child-Pugh score was 9.2 ± 1.8. Thirty-six patients (31.5%) were on beta-blockers and 40 patients (35%) were taking proton pump inhibitors (PPIs).

We followed up patients for 6 months. Four patients died (all due to acute on chronic liver failure) during this time and 8 patients had an infection (1 SBP and 7 non-SBP [3 urinary tract, 2 clostridium dificile cholitis and 2 pneumonia]). We couldn’t find any statistically significant relationship between any TLR variant genotype and the development of infections. However, 6 out of the 8 infectious episodes were present in patients bearing 2 or more TLR variant genotypes.

### TLR polymorphisms and bacterial antigen detection in patients with cirrhosis

We evaluated the incidence of 3 well-known polymorphisms in TLR-2, TLR-4 and TLR-9 in patients with cirrhosis. The distribution of genotypes and allelic frequencies in patients and controls are shown in Table 2. All variants were found to be in the Hardy–Weinberg equilibrium in the controls. Of interest, no homozygous variant genotype for TLR-4 rs4986790 was found in the cirrhotic population.

**Figure 1:**
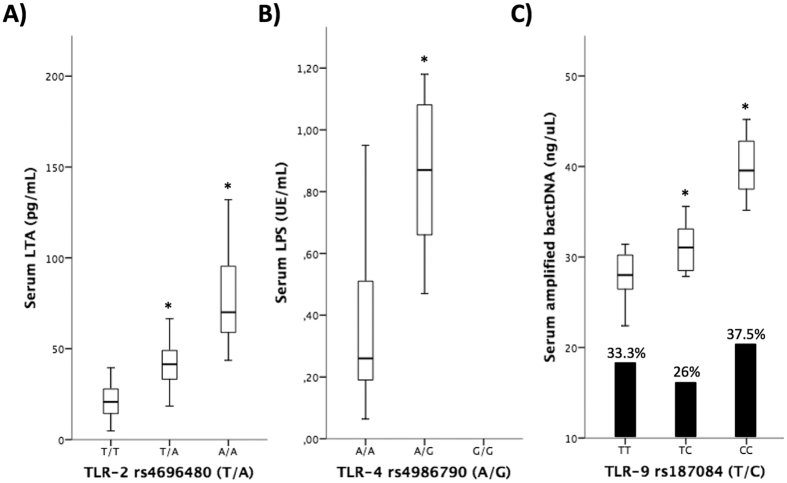
Serum levels of LTA (**A**), LPS (**B**), and amplified bacterial DNA (**C**) in blood of patients with cirrhosis distributed by TLR-2, TLR-4, and TLR-9 genotypes. The percentage of bacterial DNA translocation is also represented in panel *C*. *p < 0.05 compared to the wildtype genotypes.

Figure 1 shows the concentration of different bacterial antigens according to the polymorphisms studied in their specific receptor. As can be observed, the loss of the wild-type allele in the studied polymorphisms of TLR-2, TLR-4 and TLR-9 was associated with significantly increased serum levels of LTA, LPS and bacterial DNA, (Fig. 1A,B and C, respectively). Patients with the homozigotic variant showed the highest load of circulating bacterial antigens in blood samples.

In the case of TLR-9 rs187084, although the variant genotype was associated with an increased amplified bacterial DNA load in patients’ samples, it was not associated with an increased rate of bacterial translocation, as represented by the bars in Fig. 1C. There were not statistical differences, either, in the sequenced species from bacterial DNA between patients distributed by TLR-9 rs187084 genotype, with a similar percentage of gram-positive microorganisms in all three groups (Supplementary Table 1). Clinical and analytical characteristics of patients distributed by each TLR polymorphism genotype showed no clinical or analytical differences (data not shown).

### Soluble inflammatory response is influenced by TLR polymorphisms

**Figure 2:**
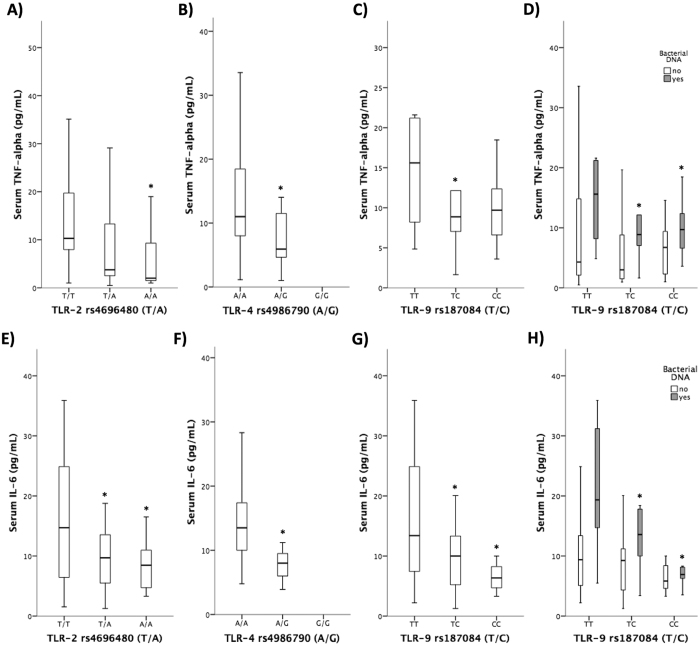
Serum levels of TNF-alpha (**A**–**C**) and IL-6 (**E**–**G**) in patients with cirrhosis distributed by TLR-2, TLR-4 and TLR-9 genotypes. (**D**,**H**) Serum levels of TNF-alpha and IL-6 in patients with cirrhosis distributed by TLR-9 genotype and the presence of bacterial DNA translocation. *p < 0.05 compared the wildtype genotypes.

Serum pro-inflammatory mediators were evaluated in all patients and compared by the genotype of studied polymorphisms. Figure 2 shows that TNF-α and IL-6 were significantly decreased in patients bearing the variant genotype of TLR-2 rs4696480 (Fig. 2A and E). When distributing the cytokine response by the variant TLR-4 rs4986790 (Fig. 2B and F) and by TLR-9 rs187084 (Fig. 2C and G) genotypes, both cytokines revealed a similar behaviour to that shown by TLR-2 rs4696480 genotype, showing significantly decreased serum levels of both mediators in patients with the variant genotypes. Patients were further distributed by the presence of detectable bacterial DNA in blood. The differences in TNF-α and IL-6 levels in patients distributed by TLR-9 rs187084 genotype were, as expected, due to those with circulating bacterial DNA in blood (Fig. 2D and H).

**Figure 3:**
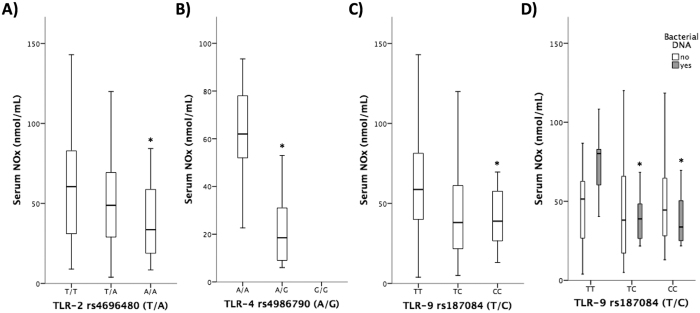
Serum levels of nitric oxide metabolites (NOx) in patients with cirrhosis distributed by TLR-2, TLR-4 and TLR-9 genotypes (**A**–**C**). (**D**) Serum levels of NOx in patients with cirrhosis distributed by TLR-9 genotype and the presence of bacterial DNA translocation. *p < 0.05 compared the wildtype genotypes.

A potent immune modulator such as NOx showed similar results to those observed for TNF-α and IL-6. The wild-type genotypes of all TLRs were significantly associated with increased serum NOx levels (Fig. 3A–C). As shown for the cytokine pattern, the differences in NOx levels in TLR-9 rs187084 genotyped patients were associated with circulating bacterial DNA in blood (Fig. 3D).

We further analysed the inflammatory milieu in the serum of patients grouped by the number of variant TLR genotypes. As observed in Table 3, the accumulation of variant TLR genotypes was associated with significantly decreased pro-inflammatory cytokine and NOx levels.

### Immunodeficiency in decompensated cirrhosis is associated with variant TLR-2, TLR-4 and TLR-9 genotypes

We evaluated the *in vitro* response to specific TLR ligands to confirm the downregulation of the inflammatory milieu in patients and donors bearing variant TLR genotypes. TNF-α, IL-6 and IL-10 levels in patients’ PMNs supernatants were significantly upregulated in all cases after stimulation with Pam3-Cys, LPS and CpGs compared with resting conditions. However, TNF-α and IL-6 levels were significantly lower and IL-10 was significantly higher in response to TLR-specific ligands in patients with variant TLR-2 rs4696480, TLR-4 rs4986790 and TLR-9 rs187084 genotypes compared with those bearing the wild-type alleles (Table 4). Results on donors are shown in Table 5 and, as well as for patients, the presence of variant genotypes in all studied TLRs was associated with significantly reduced pro-inflammatory cytokine levels and increased IL-10 compared with the wild-type genotypes.

**Figure 4:**
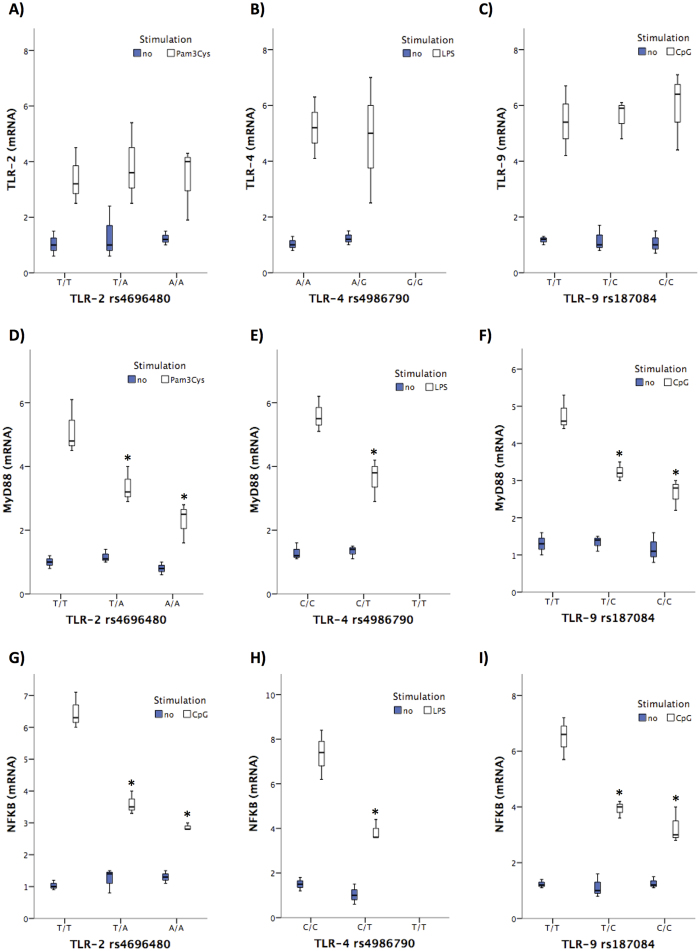
Gene expression levels of TLR-2, TLR-4, TLR-9, MyD88 and NFkB in cultured PMNs from patients with cirrhosis distributed by TLR-2 rs4696480 (**A**,**D**,**G**), TLR-4 rs4986790 (**B**,**E**,**H**) and TLR-9 rs187084 (**C**,**F**,**I**) genotypes. *p < 0.05 compared with non-stimulated conditions.

Interestingly, the gene expression levels of variant TLR-2 (Fig. 4A), TLR-4 (Fig. 4B), and TLR-9 (Fig. 4C) were not significantly different in response to their specific ligands compared with those observed in cells from patients with the wild-type alleles. However, when evaluating the gene expression levels of downstream signalling molecules, the presence of studied SNPs in TLR-2, TLR-4 and TLR-9 were associated with a significant decrease in MyD88 (Fig. 4D–F) and NFkB (Fig. 4G–I) compared with cells from patients with wildtype alleles after stimulation with their respective specific ligands. Statistical correlations between MyD88 and NFkB gene expression levels and the concentration of *in vitro* secreted cytokines are shown in Table 6.

## Discussion

In this study, we demonstrate that variant genotypes in TLR-2, TLR-4 and TLR-9 genes are associated with an increased bacterial antigen burden and a decreased pro-inflammatory cytokine profile in blood of patients with cirrhosis and non-infected AF, suggesting that these genetic variants may compromise bacterial antigen interaction with their specific receptors and limit the innate soluble inflammatory response in these patients. This genetically derived immunodeficiency might have consequences in bacterial antigen clearance and contribute to the clinically relevant complications that are frequently developed in patients with cirrhosis.

A compromised immunological status is one of the three main aspects classically proposed for the development of bacterial antigen translocation episodes in cirrhosis, which in turn, is proposed to be either responsible or a contributor to life-threatening complications in these patients such as SBP, hepatic encephalopathy or haemodynamic disturbances. TLRs take part in the specific recognition of antigenic molecules and trigger early events in the immunological response. We have evaluated the effect of TLR2 rs4696480, TLR4 rs4986790 and TLR9 rs187084 polymorphisms in the bacterial antigen load and the pro-inflammatory mediator levels in the blood of a consecutive series of patients with cirrhosis and AF.

We first show that TLR variant genotypes are associated with significantly increased serum levels of their specific antigenic ligands (LTA, LPS and bacterial DNA, respectively). This would be in line with previous findings showing an increased risk of SBP in patients with cirrhosis bearing TLR-2 polymorphisms^13^ or the predisposition to infections in cirrhotic patients with a variant TLR-4 genotype^14^. Interestingly, although the rate of bacterial DNA translocation is not increased in patients with a variant TLR9 genotype, there is a significant increment in the amount of amplified bacterial DNA in these patients compared with those bearing the wild-type TLR-9 genotype. This is relevant, as it has been proven that the grade of soluble inflammatory response is significantly affected by bacterial DNA concentrations in patients with cirrhosis^18^.

However, an important result of the study is that evaluated TLR variant genotypes show a reduced secretion of pro-inflammatory mediators in the serum of cirrhotic patients, despite their significantly increased antigenic burden (Fig. 2). Moreover, the presence of several variant TLR polymorphisms is associated with a progressive, significantly reduced inflammatory milieu. To confirm our results, we evaluated the soluble response to TLR-specific ligands in *in vitro* cultured neutrophils from cirrhotic patients and donors with different TLR genotypes. As shown *in vivo*, although TLR stimulation resulted in significantly increased supernatant levels of pro-inflammatory cytokines compared with resting cells, the variant genotypes showed a significantly decreased ability to induce TNF- and IL-6 in response to Pam3-cys, LPS and CpGs compared with wild-type TLR cells, both in patients (Fig. 4) and donors (Table 3), suggesting that the impact of studied polymorphisms on the immune system is independent of the pathological context.

These results would restrict findings in previous studies to wild-type TLR-genotyped patients and they would suggest either a lack of the antigenic ligand recognition by TLR or a TLR variant genotype-induced breakdown in the pro-inflammatory signalling pathway. Our results *in vitro* show no differences in TLR gene expression levels, suggesting a competent stimulation of these receptors despite their polymorphic regions (Fig. 4A–C). On the contrary, downstream signalling molecules were reduced in cells from TLR variant *vs* wildtype genotyped patients (Fig. 4D–I), supporting the second hypothesis. Either way, as a result, bacterial antigenic internalization and/or cellular signalling events would be compromised, leading to serum free antigen accumulation and low pro-inflammatory cytokine levels. In fact, hyporresponsiveness to LPS has been described in the presence of TLR-4 variant genotypes^15^ and TLR-4 expression downregulation^19^. This resultant “tolerant state” might account for the increased risk of infections commented before.

The mechanisms by which the TLR polymorphisms may condition receptor are not fully elucidated. Several controversial examples were nicely reviewed in the recent past by *Medveded A*^20^. For example, the R753Q TLR-2 polymorphism does not significantly affect TLR2 expression, but induces the suppression of NF-kB activation and cytokine expression by affecting the electrostatic potential of the TIR domain^21^. Other TLR2 variants, such as P681H, do not change wild-type TLR-2 expression levels^22^, but it is associated with slower rates of internalization from the cell surface to endosomes^23^. The D299G TLR-4 variant is associated with decreased TLR-4 expression levels in response to LPS^15^. In contrast, others have reported no changes in expression levels of WT, D299G, or T399I TLR4 variants in HEK293T/CD14/MD2 transfectants, but a compromised ability to elicit NFkB activation in response to LPS in the TLR-4 variants^24^,^25^. In fact, despite similar TLR-4 expression levels, PMNs from individuals expressing the D299G TLR4 variant showed reduced phosphorylation of IkB-a and secretion of IL-12 p17 upon stimulation with LPS compared with cells expressing WT TLR4^26^. D299G polymorphism has also been described to inhibit LPS-induced association of TLR-4 with adapters MyD88 and TRIF, resulting in suppressed activation of the transcription factors NF-kB and IRF-3, p38 MAPK phosphorylation, and induction of MyD88- and TRIF-dependent cytokines^27^.

From a clinical standpoint, TLR polymorphisms have widely been associated with human disease^28^. Particularly, bacteria-related complications and the associated inflammatory response are relevant to the TLR polymorphism evaluation, as several members of this family are specific receptors of their products. In fact, previously TLR-2 and TLR-4 polymorphism have been associated with spontaneous bacterial peritonitis or bacteremia in cirrhotic patients^13^,^14^ and the immunological hyporesponsiveness to LPS in TLR-4 variants has been previously reported^15^. As an explanation for those associations, we now demonstrate that the circulating antigen burden is significantly increased in variant-TLR cirrhotic patients. As the translocation of bacterial products is frequent and recurrent in cirrhosis, it is likely that genetically deficient TLR signalling might be involved and, therefore, provide new targets for treating bacteria-derived complications in cirrhosis.

In summary, polymorphisms in TLR-2, TLR-4 and TLR-9 genes are associated with an increased bacterial antigen burden and a deficient pro-inflammatory cytokine response in patients with cirrhosis. Both aspects may delay bacterial antigen clearance in blood and contribute to clinically relevant bacteria-derived complications in patients with cirrhosis.

## Patients and Methods

### Patients and study design

We conducted a prospective observational study in patients with cirrhosis and non-infected ascitic fluid (AF) consecutively admitted or followed at our Hospital from January 2014 to January 2016.

Exclusion criteria were the presence of spontaneous bacterial peritonitis (SBP) or infections, multinodular hepatocellular carcinoma, portal thrombosis, alcoholic hepatitis, previous liver transplantation or previous transjugular intrahepatic portosystemic shunt. SBP was defined as the presence of >250 polymorphonuclear cells/μL in AF. The Ethics committee of Hospital General Universitario de Alicante approved the study protocol, and all patients gave informed consent to participate in the study. All methods described herein were performed in accordance with the relevant guidelines and regulations.

Blood and AF were obtained from all patients at admission and analyzed for routine biochemical and cytological studies. Blood and AF cultures were performed in all cases. Aliquots of blood and AF were inoculated under aseptic conditions in sterile, rubber-sealed Vacutainer SST II tubes (BD Diagnostics, Belgium) that were never exposed to free air.

Patients were followed-up for 6 months. Incidence of infections, successive hospitalizations and mortality were registered.

### TLR polymorphisms genotyping

The studied SNPs were TLR4 rs4986790 on chromosome 9q33.1, TLR9 rs187084 on chromosome 3p21.2 and TLR2 rs4696480 on chromosome 4q31.3. Genotyping was performed in genomic DNA extracted from peripheral blood samples by using the QIAamp DNA Blood Mini Kit (Qiagen) according to manufacturer’s recommendations. Partial amplification of the genes containing the different polymorphisms was performed using specific primers as follows: TLR4 rs4986790 forward 5′-CTACCAAGCCTTGAGTTTCTAG-3′, reverse 5′-AAGCTCAGATCTAAATACCT-3′; TLR9 rs187084 forward 5′-CATTCATTCAGCCTTCACTC-3′, reverse: 5′-ATGTGCTGTTCCCTCTGC-3′; TLR2 rs4696480 forward 5′-GGGACAAGAATAAAGTACATAGTTG-3′, reverse 5′-GGCTGTACCCTCATAAATGGA-3′. PCR product sizes (110 bp, 419 bp and 297 bp, respectively) were purified using ExoSAP-IT PCR Product Cleanup (Affymetrix). The incidence of polymorphisms was detected by nucleotide sequencing of PCR products using the same primers as for the amplification. The sequencing process was performed by Secugen SL. The results were analysed with FinchTV software version 1.5 (Geospiza). The incidence of all three polymorphisms in control population was obtained from The 1000 Genomes Project Consortium^29^ (www.1000genomes.org).

### Bacterial antigen measurement in patients’ samples

Samples and reagents were handled in an airflow chamber and processed with pyrogen-free material tested by manufacturers. To determine lipoteichoic acid (LTA), the specific ligand of TLR-2, a Human lipoteichoic acid elisa kit (Abbexa Ltd., Cambridge, UK) was used according to manufacturer’s instructions. A quantitative chromogenic limulus amebocyte lisate (LAL) test (BioWhittaker, Nottingham, UK) was followed to evaluate endotoxin levels in blood and AF samples as previously described^30^. To detect the presence of bacterial DNA fragments in blood and AF, a broad-range polymerase chain reaction (PCR) was performed according to the methodology described elsewhere^31^. PCR amplicons were loaded onto DNA Laboratory-on-chips (Agilent Technologies, Palo Alto, CA) and analyzed with an Agilent 2100 BioAnalyzer.

### Inflammatory mediators quantification

Enzyme-linked immunosorbent assays (ELISAs) for the quantitative measurement of TNF-α and IL-6 levels were carried out in serum samples of patients by handling Human Quantikine kits (R&D Systems, Minneapolis, MN), according to manufacturer’s instructions. All samples were tested in triplicate and read in a microplate reader. Lower limits of detection of all cytokine assays were 5–8 pg/mL. Standard curves were generated for each plate, and the average zero standard optical densities were subtracted from the rest of standards, controls, and samples to obtain a corrected concentration for both cytokines.

The sum of the NO metabolites nitrite (NO_2_^−^) and nitrate (NO_3_^−^) is widely used as an index of NO generation^32^ and expressed as NOx levels (nmol/ml). NOx levels were calculated by measuring conversion of NO_3_^−^ to NO_2_^−^ by the enzyme nitrate reductase using an ELISA assay (R&D Systems, Minneapolis, USA). All samples were tested in duplicate and values were corrected by running samples with culture media without cells to assess background NOx levels.

### Cell cultures

Human peripheral blood polymorhonuclear cells (PMNs) were isolated from patients and donors by Polymorph Prep solution (Axis-Shield, Oslo, Norway). Cells were washed twice with freshly made phosphate-buffered saline (PBS) at 4 °C and viability was evaluated by trypan blue (Sigma, Madrid, Spain). Cells were cultured in phenol red free RPMI 1640 medium (Gibco BRL, Life Technologies, Paisley, UK) supplemented with L-glutamine, antibiotics and 10% FBS at a concentration of 10^6^ cells/ml in presence of no stimulus, CpGs ODN 2395 (20 ng/ml) (InvivoGen, San Diego, CA), LPS (100 ng/ml) (*E. coli* serotype 0111:B4; Sigma) or Pam3CSK4 (100 ng/ml) (InvivoGen) during 24 hours. After that period, all supernatants and pellets were collected and stored at −20 °C.

### Quantitative PCR analysis

Total RNA was extracted using QIAamp RNA Blood Mini Kit (QIAGEN) and quantitative PCRs were performed in order to evaluate the expression of Toll-like receptors studied, as well as the key genes in their signaling cascade. The reactions were performed in a 12.5 uL PCR mixture using qScript One-Step SYBR Green RT-qPCR (Quanta BioScience, Gaithesburg, Maryland). The specific primers used were: 5′ TGTGACCGCAATGGTATCTG 3′ (forward) and 5′TGTTGTTGGACAGGTCAAGG 3′ (reverse) for TLR2, 5′ TCCATAAAAGCCGAAAGGTG 3′ (forward) and 5′ GATACCAGCACGACTGCTCA 3′ (reverse) for TLR4, 5′ GGGAGCTACTAGGCTGGTATAAAAATC 3′ (forward) and 5′ GCTACAGGGAAGGATGCTTCAC 3′ (reverse) for TLR9, 5′ GGACCCAGCATTGAGGAG 3′ (forward) and 5′ ACAGCGGCCACCTGTAAA 3′ (reverse) for MyD88, 5′ TCATGAAGAAGAGTCCTTTCAGC 3′ (forward) and 5′ CTGGCTTGGGGACAGAAG 3′ (reverse) for nF-KB, 5′ GACTCCATCTTGGCTGTGA 3′ (forward) and 5′ TGATTTCTGCTCTGACAACCT 3′ (reverse) for IFN-α, 5′ AGGACAGGATGAACTTTGAC 3′ (forward) and 5′ TGATAGACATTAGCCAGGAG 3′(reverse) for IFN-β. Relative mRNA levels were calculated by normalizing to an endogenous reference gen (GAPDH).

### Statistical analysis

Continuous variables are reported as mean ± standard deviation (or as median [25th–75th percentiles] in Figures) and categorical variables as frequency or percentages. Quantitative data were analysed using the Mann-Whitney U test for simple comparisons or the Kruskal-Wallis test followed by pairwise comparisons using the Mann-Whitney U test with the post-hoc Bonferroni correction for multiple comparisons. Differences in qualitative variables were analysed using the χ2 test. Bivariate correlations between continuous variables were calculated using the Spearman test. All reported *p* values are 2-sided, and *p* values lower than 0.05 were considered to indicate significance. All calculations were performed using the IBM SPSS Statistics 19.

## Additional Information

**How to cite this article**: Piñero, P. *et al*. Toll-like receptor polymorphisms compromise the inflammatory response against bacterial antigen translocation in cirrhosis. *Sci. Rep.* **7**, 46425; doi: 10.1038/srep46425 (2017).

**Publisher's note:** Springer Nature remains neutral with regard to jurisdictional claims in published maps and institutional affiliations.

## Supplementary Material

Supplementary Information

## Figures and Tables

**Table 1:** Clinical and analytical characteristics of patients.

Age	(years)	62 ± 10
Gender	(male/female)	76/38
Etiology	Alcohol	51 (44.7%)
	HCV	32 (28.1%)
	Alcohol + HCV	19 (16.7%)
	Alcohol + HBV	3 (2.7%)
	Other	9 (7.8%)
Previous episodes of ascites	n (%)	48 (42.1%)
Previous episodes of encephalopahty	n (%)	14 (12.3%)
Previous episodes of variceal bleeding	n (%)	20 (17.5%)
SBP (previous 6 months)	n (%)	2 (1.7%)
Infections other than SBP (previous 6 months)	n (%)	10 (8.8%)
Child-Pugh Category	A/B/C	0/66/48
Child-Pugh mean score		9.2 ± 1.8
MELD mean score		12.5 ± 3.7
Use of beta-blockers	n (%)	31 (27.2%)
Use of PPIs	n (%)	34 (29.8%)
Mean arterial pressure	(mmHg)	85.5 ± 8.5
Heart rate	(bpm)	79 ± 12
Bilirubin	(mg/dL)	3.1 ± 2.4
Albumin	(mg/dL)	2.6 ± 0.7
AST	(IU/L)	65.3 ± 45.7
ALT	(IU/L)	37.5 ± 28.8
Quick	(%)	67 ± 17
INR		1.4 ± 0.3
Serum creatinine	(mg/dL)	0.96 ± 0.4
Serum sodium	(mEq/L)	138.7 ± 4.6
Serum potassium	(mEq/L)	4.4 ± 0.6
Platelets	(/mm3)	120,474 ± 60,749
Blood WBC	(/mm3)	5645 ± 2627

SBP: Spontaneous bacterial peritonitis; PPIs: protom-pump inhibitors; WBC: white blood cells.

**Table 2:** Genotype and allele frequencies for studied TLR polymorphisms.

	Genotype n (%) patients/controls	Heterozygous	Homozygous variant	Variant allele frequency (%)
	Homozygous wild type			
TLR-2 rs4696480 (T/A)	25 (21.9%)/121 (24.1%)	55 (48.2%)/243 (48.3%)	34 (29.8%)/139 (27.6%)	54.9/51.8
TLR-4 rs4986790 (A/G)	98 (85.9%)/448 (89.1%)	16 (14.1%)/52 (10.3%)	0 (0%)/3 (0.6%)	5.0/5.0
TLR-9 rs187084 (T/C)	44 (38.5%)/161 (32.0%)	53 (46.4%)/254 (50.5%)	17 (14.9%)/88 (17.5%)	37.0/42.0

**Table 3:** Serum cytokine and nitric oxide levels in patients grouped by the number of variant TLR genotypes.

		TNF-α (pg/mL)	IL-6 (pg/mL)	NOx (nmol/mL)
Wildtype TLRs	n = 16	16.5 ± 7.9	18.4 ± 9.1	67.4 ± 18.5
One variant TLR	n = 46	11.5 ± 6.8*	13.7 ± 7.5	56.0 ± 23.7
Two variant TLRs	n = 42	7.8 ± 3.8*	10.3 ± 3.9*	55.8 ± 19.5*
Three variant TLRs	n = 10	2.8 ± 2.5*	8.5 ± 1.4*	33.6 ± 17.1*

TNF-α: tumor necrosis factor alpha; IL: interleukin; NOx: nitric oxide metabolites; *p < 0.01 compared with wildtype TLRs.

**Table 4:** Secreted levels of cytokines in the supernatants of cultured PMNs from patients distributed by the genotype of TLR polymorphisms.

	Total n = 114	TNF-α (pg/mL)		IL-6 (pg/mL)		IL-10 (pg/mL)	
		Not stimulated	Stimulated^#^	Not stimulated	Stimulated^#^	Not stimulated	Stimulated^#^
TLR2 rs4696480	T/T (n = 25)	9.0 ± 2.7	270.0 ± 69.3*	26.8 ± 8.6	522.0 ± 34.6*	5.1 ± 3.2	5.6 ± 3.9
	T/A (n = 55)	8.6 ± 2.5	66.3 ± 17.8^*,$^	26.2 ± 4.9	446.3 ± 12.2^*,$^	3.9 ± 3.2	10.8 ± 4.9^*,$^
	A/A (n = 34)	6.9 ± 3.1	29.7 ± 6.1^*,$^	30.3 ± 3.8	332.6 ± 28.4^*,$^	4.3 ± 2.6	16.8 ± 7.2^*,$^
TLR4 rs4986790	A/A (n = 98)	8.9 ± 5.8	378.3 ± 41.6*	22.2 ± 7.9	360.7 ± 39.5*	4.0 ± 3.5	8.5 ± 5.3*
	A/G (16)	8.5 ± 3.3	338.6 ± 34.4*	26.8 ± 4.8	353.3 ± 43.9^*,$^	4.2 ± 2.9	16.3 ± 8.1^*,$^
TLR9 rs187084	T/T (n = 44)	8.9 ± 5.5	77.3 ± 37.5*	28.6 ± 6.5	422.0 ± 23.4*	4.3 ± 3.8	10.1 ± 6.5*
	T/C (n = 53)	10.8 ± 3.3	39.0 ± 14.1^*,$^	27.1 ± 4.4	338.0 ± 41.1^*,$^	3.8 ± 2.9	15.4 ± 6.6^*,$^
	C/C (n = 17)	7.3 ± 3.0	19.2 ± 2.6^*,$^	28.0 ± 2.8	213.0 ± 62.9^*,$^	3.7 ± 3.6	19.6 ± 7.5^*,$^

^#^Stimuli: Pam3CSK4 (100 ng/ml) for TLR-2; LPS (100 ng/ml) for TLR-4; and CpGs ODN 2395 (20 ng/ml) for TLR-9. *p < 0.01 compared with the unstimulated condition; ^$^p < 0.01 compared with the stimulated wildtype TLR genotype.

**Table 5:** Secreted levels of cytokines in the supernatants of cultured PMNs from donors distributed by the genotype of TLR polymorphisms.

	Total n = 20	TNF-α (pg/mL)		IL-6 (pg/mL)		IL-10 (pg/mL)	
		Not stimulated	Stimulated^#^	Not stimulated	Stimulated^#^	Not stimulated	Stimulated^#^
TLR2 rs4696480	T/T (n = 8)	6.2 ± 3.3	186.4 ± 74.6*	8.5 ± 5.3	410.0 ± 224.2*	3.5 ± 3.4	12.6 ± 6.8*
	T/A (n = 6)	5.2 ± 2.8	88.2 ± 32.8^*,$^	7.2 ± 4.8	250.4 ± 119.6^*,$^	5.3 ± 4.1	19.4 ± 9.2^*,$^
	A/A (n = 6)	6.1 ± 3.1	35.1 ± 20.6^*,$^	9.4 ± 6.2	100.8 ± 80.4^*,$^	3.4 ± 2.7	25.6 ± 12.4^*,$^
TLR4 rs4986790	A/A (n = 13)	7.3 ± 3.6	364.6 ± 104.6*	7.7 ± 5.4	450.4 ± 168.3*	3.5 ± 2.6	9.4 ± 3.8*
	A/G (7)	5.8 ± 4.1	188.6 ± 75.7^*,$^	10.1 ± 7.3	286.5 ± 104.7^*,$^	4.1 ± 3.2	15.8 ± 6.2^*,$^
TLR9 rs187084	T/T (n = 7)	6.6 ± 3.2	72.2 ± 26.5*	6.9 ± 3.5	402.6 ± 175.4*	4.9 ± 3.3	13.5 ± 8.5*
	T/C (n = 7)	5.5 ± 3.5	45.3 ± 22.1^*,$^	7.3 ± 4.5	283.3 ± 98.8^*,$^	3.9 ± 2.4	18.6 ± 10.1*
	C/C (n = 6)	5.2 ± 3.0	19.2 ± 8.8^*,$^	6.6 ± 3.8	137.5 ± 77.3^*,$^	3.6 ± 3.0	26.6 ± 11.2^*,$^

^#^Stimuli: Pam3CSK4 (100 ng/ml) for TLR-2; LPS (100 ng/ml) for TLR-4; and CpGs ODN 2395 (20 ng/ml) for TLR-9. *p < 0.01 compared with the unstimulated condition; ^$^p < 0.01 compared with the stimulated wildtype TLR genotype.

**Table 6:** Correlations between *in vitro* secreted cytokine levels and TLR signalling molecules.

	Secreted TNF-α (pg/10^6^ cells)	Secreted IL-6 (pg/10^6^ cells)	Secreted IL-10 (pg/10^6^ cells)
*MyD88* mRNA	r = 0.817; p < 0.001	r = 0.834; p < 0.001	r = −0.616; p < 0.001
*NFkB* mRNA	r = 0.789; p < 0.001	r = 0.815; p < 0.001	r = −0.685; p < 0.001

TNF-α: tumor necrosis factor alpha; IL: interleukin; MyD88: Myeloid differentiation primary response gene 88; NFkB: nuclear factor kappa B.
