# Supplementary material for: Toll-like receptor polymorphisms compromise the inflammatory response against bacterial antigen translocation in cirrhosis
Source: Sci Rep. 2017 Apr 18;7:46425. doi: 10.1038/srep46425 (PMC5394473; doi:10.1038/srep46425)
Supplement: Supplementary Information [file srep46425-s1.doc]

**Title:** Toll-like receptor polymorphisms compromise the inflammatory response against bacterial antigen translocation in cirrhosis.

**Authors**: Paula Piñero^1^, Oriol Juanola^1^, Esther Caparrós^2^, Pedro Zapater^3,4^, Paula Giménez^4^, José M González-Navajas^1,4^, José Such^5^, Rubén Francés^1,2,4 *^.

**Supplementary Table 1.** Identified bacterial species from bacterial DNA detected in patients distributed by TLR-9 rs187084 genotype.
